# Development and internal validation of a preliminary cumulative odontogenic risk categorization score for identifying factors associated with maxillary sinus membrane thickening: a retrospective cross-sectional study

**DOI:** 10.1186/s12903-026-09030-x

**Published:** 2026-06-25

**Authors:** Yu-Ming Kuo, Hsin-Yi Huang, Chieh-An Yi, Yi-Chun Lin, Ya-Chi Chen, Hsuan-Hung Chen

**Affiliations:** 1 Division of Periodontics, Department of Stomatology, Taipei Veterans General Hospital, No.201, Sec. 2, Shipai Rd., Beitou District, Taipei, 11217 Taiwan; 2 Department of Information Management, Taipei Veterans General Hospital, Taipei, Taiwan; 3 Big Data Center, Taipei Veterans General Hospital, Taipei, Taiwan; 4 School of Dentistry, National Yang Ming Chiao Tung University, Taipei, Taiwan

**Keywords:** Cone-beam computed tomography, Maxillary sinus, Periapical periodontitis, Periodontitis, Scoring system, Associated factors, Schneiderian membrane

## Abstract

**Background:**

Periodontal bone loss (PBL) is a traditional metric for periodontitis, but its ability to reflect the total regional inflammatory burden in the posterior maxilla is limited. This study aimed to develop and internally validate a preliminary cumulative odontogenic score (COS) by integrating periodontal and endodontic parameters to characterize their combined association with maxillary sinus membrane thickening (SMT).

**Methods:**

This retrospective cross-sectional study analyzed 1,056 dentate maxillary sinuses from 562 patients. Odontogenic variables, including PBL severity, endodontic treatment history, and periapical lesions, were assessed using cone-beam computed tomography (CBCT). Multivariable logistic regression using generalized estimating equations (GEE) was performed to identify independent factors associated with SMT (> 2 mm). A COS (range 1–7) was derived from weighted β regression coefficients. Classification performance was evaluated through ROC analysis, calibration plots, and bootstrapping (1,000 resamples).

**Results:**

SMT was prevalent in 46.3% of sinuses. PBL, periapical lesions, and endodontic treatment were all independent contributors to SMT (*p* < 0.001). The COS demonstrated significantly superior classification performance compared to PBL alone (AUC 0.718 vs. 0.602; *p* < 0.001) and exhibited adequate calibration (slope = 1.00). Internal validation via bootstrapping confirmed the stability of the associations. Each one-point increase in the COS was associated with a 30% increase in the odds of SMT. Using a clinically relevant threshold of COS > 3, the prevalence of SMT in the high-COS group was nearly double that of the low-COS group (59.9% vs. 30.5%).

**Conclusions:**

Integrating periodontal and endodontic conditions into a unified scoring framework provides a more adequate characterization of the dental-sinus association than individual parameters. The internally validated COS offers a structured approach for preoperative radiographic evaluation and can function as a preliminary cross-sectional risk categorization score to characterize interdisciplinary associations in the posterior maxilla.

## Background

Periodontal inflammation is traditionally characterized by periodontal bone loss (PBL), a hallmark of cumulative structural destruction. Under the 2018 World Workshop classification, staging is primarily dictated by the severity of clinical attachment and alveolar bone loss [1, 2]. While PBL is an established metric of historical tissue breakdown, it may not sufficiently capture the total regional inflammatory burden in the posterior maxilla, where proximity to adjacent structures necessitates a more localized assessment [3].

Current evidence suggests that sinus membrane thickening (SMT) often represents a reactive mucosal response to odontogenic stimuli rather than an isolated sinonasal phenomenon [4, 5]. Periapical pathology and PBL severity have both been linked to increased SMT prevalence [6–10], suggesting the sinus membrane functions as a sensitive biological indicator of adjacent dental health.

However, periodontal destruction rarely manifests in isolation. Endodontic treatment history and periapical inflammation frequently coexist with periodontal breakdown, particularly in endodontic–periodontal lesions [11, 12]. Even in root-filled teeth, persistent apical inflammatory activity can exert continuous influence on the sinus membrane [13]. Consequently, relying solely on PBL likely underestimates the cumulative odontogenic inflammatory burden.

This diagnostic limitation impacts the interdisciplinary management of periodontists, endodontists, and oral surgeons [14]. A framework integrating multiple odontogenic inflammatory sources may provide a more comprehensive approach to regional characterization, especially for procedures like sinus floor elevation. Transitioning from subjective observation to objective categorization is essential for standardized evaluation.

Therefore, the aim of this retrospective study was to develop and internally validate a preliminary cumulative odontogenic score (COS) framework—incorporating PBL, endodontic treatment history, and periapical pathology—to evaluate its clinical relevance for identifying factors associated with SMT. This study seeks to provide a structured risk categorization score for characterizing regional inflammatory interactions in the posterior maxilla.

## Methods

### Study design

This retrospective cross-sectional study analyzed clinical and radiographic records of patients who underwent cone-beam computed tomography (CBCT) for implant-related evaluation at the Department of Stomatology, Taipei Veterans General Hospital between 2014 and 2021. The primary aim was to develop and internally validate the COS for characterizing associations with SMT.

### Participants and eligibility criteria

**Fig. 1:**
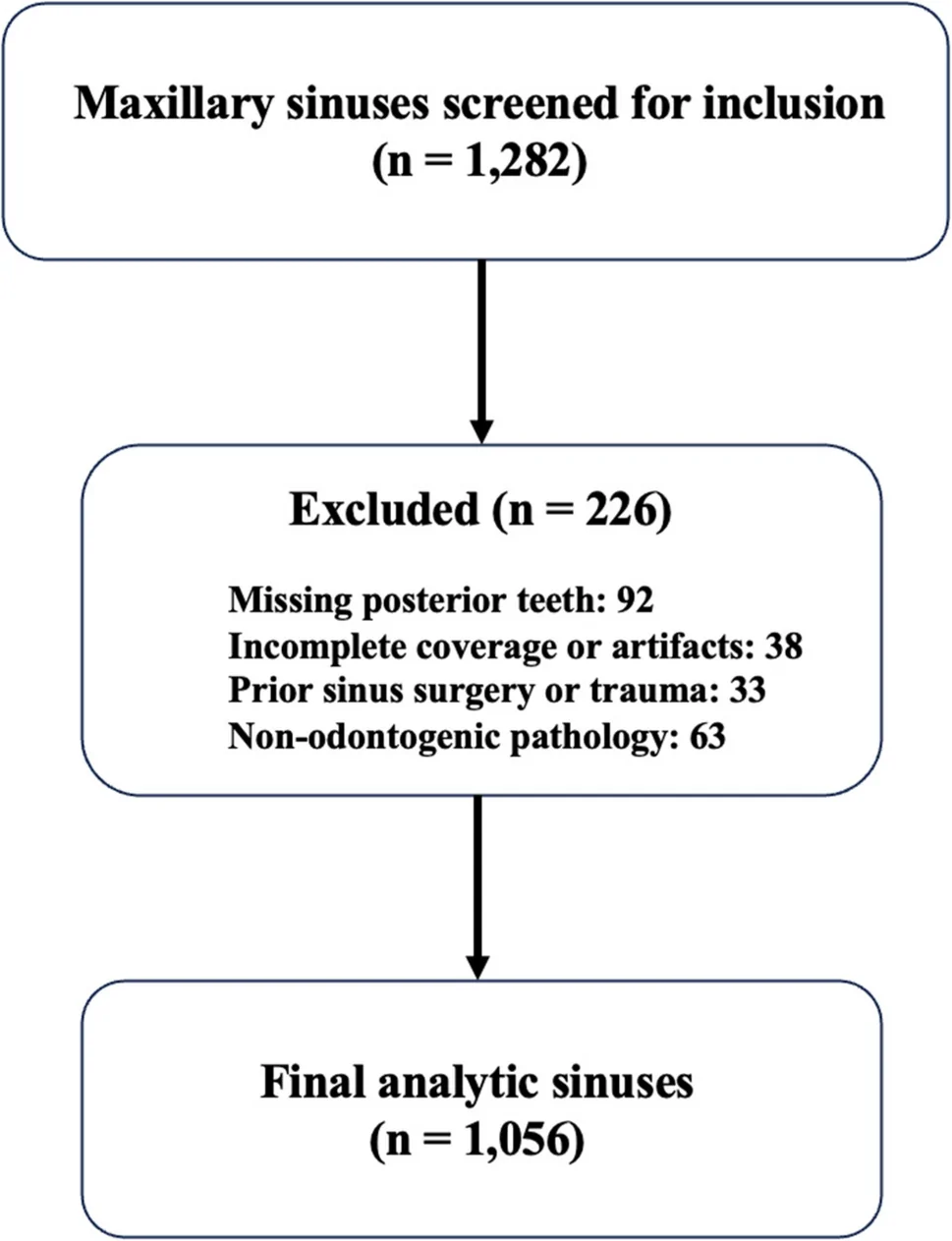
Study flow diagram of the sinus selection process. The diagram illustrates the consecutive enrollment and exclusion criteria applied at the sinus level. Out of 1,282 maxillary sinuses screened between 2014 and 2021, 226 were excluded based on specific radiographic and clinical criteria, resulting in a final analytical sample of 1,056 sinuses from 562 patients

To minimize selection bias, a consecutive sampling strategy was employed. Patients were eligible for inclusion if they were older than 20 years and had undergone CBCT examination covering the posterior maxilla for preoperative assessment at the same institute during the study period. For the primary analysis of odontogenic factors, only dentate maxillary sinuses were included. A sinus was classified as dentate if at least one posterior maxillary tooth (first premolar through third molar) was present beneath the sinus floor. Sinuses were excluded based on the following criteria: (1) CBCT images with incomplete coverage of the inferior third of the maxillary sinus or significant image artifacts precluding accurate diagnostic analysis; (2) a history of maxillary sinus augmentation or midfacial trauma; (3) prior surgical treatment for sinonasal pathology or the presence of clinically significant obstructive sinonasal lesions (e.g., tumors or large polyps); and (4) radiographic evidence of primary non-odontogenic sinus infection (e.g., pansinusitis or air-fluid levels) at the time of imaging. These criteria ensured that the study population was limited to patients seeking dental treatment without active symptomatic sinonasal complaints. The final analytic database was complete, as cases with missing clinical or radiographic variables were removed during initial screening. Due to the retrospective, cross-sectional design, all predictors and the outcome (SMT) were assessed concurrently on the same CBCT volumes. A complete-case analysis was performed without the need for data imputation. The detailed selection process and sinus-level attrition are illustrated in Fig. 1.

### Ethical considerations

This study protocol was reviewed and approved by the Institutional Review Board of Taipei Veterans General Hospital (IRB-TPEVGH No. 2022-02-015AC). The study was conducted in accordance with the Declaration of Helsinki of 1975, as revised in 2013. Reporting followed the Strengthening the Reporting of Observational Studies in Epidemiology (STROBE) guidelines [15]. The requirement for informed consent was formally waived by the Institutional Review Board of Taipei Veterans General Hospital due to the retrospective nature of the study and the use of de-identified, anonymized data.

### Radiographic analysis and characterization of SMT

CBCT imaging was performed using a standardized protocol (NewTom 5G, QR, Verona, Italy) for preoperative implant assessment (16 mA, 110 kV, field of view of 12 × 8 cm, exposure time 7.3 s, slice thickness 1 mm). Radiographic evaluations were conducted using dedicated imaging software (SmartIris, TEDPC UltraQueryEx 1.0.5.3, Taipei, Taiwan).

**Fig. 2:**
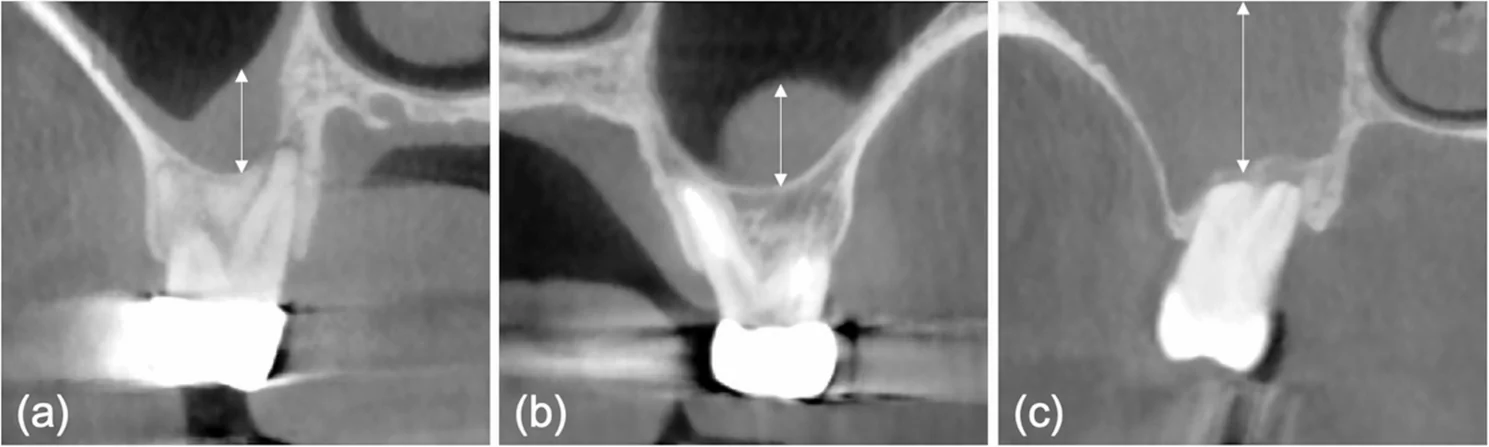
Radiographic assessment of maxillary sinus membrane thickening (SMT). **a** Sagittal CBCT view showing a flat SMT pattern (> 2 mm). **b** Polypoid morphology characterized by a dome-shaped lesion. **c** Mucocele-like appearance with complete sinus opacification. The measurement representsthe maximum thickness perpendicular to the bony wall

To identify the presence of membrane thickening, the maxillary sinus membrane was serially evaluated in sagittal CBCT images spanning the mesial to distal extent of each sinus (Fig. 2). SMT was defined as a membrane thickness > 2 mm, measured perpendicular to the adjacent bony wall at the point of maximum thickness [16].

The morphological pattern of SMT was categorized based on established radiographic appearances.

- Flat: Shallow membrane thickening without a well-defined outline.
- Polypoid: Dome-shaped lesion with a well-defined outline rising at an angle > 30° from the sinus floor or wall.
- Mucocele-like: Complete or near-complete opacification of the maxillary sinus [6, 17].

### Odontogenic conditions in the posterior maxilla

Odontogenic inflammatory sources were assessed on reconstructed CBCT images, encompassing the region from the first premolar through the third molar.

Endodontic status was characterized by two variables: (1) Endodontic treatment history, identified by the presence of radiopaque canal filling material; and (2) Periapical lesions, defined as radiolucencies associated with loss of the lamina dura. Both variables were coded as positive if at least one adjacent tooth demonstrated the corresponding finding, serving as indicators of periradicular inflammatory reservoirs.

Periodontal status was assessed using PBL as a metric for chronic structural destruction. PBL was classified into coronal-third (reference), middle-third, and apical-third groups based on the vertical extent of bone loss relative to root length. The most severe PBL associated with each sinus was used for analysis to reflect the maximum regional periodontal inflammatory burden.

### Statistical analysis

#### Descriptive and reliability analysis

Statistical analyses were initiated using IBM SPSS Statistics, version 25.0 (IBM Corp., Armonk, NY, USA) for descriptive statistics and univariate comparisons. Continuous variables were summarized as means ± standard deviations, and categorical variables as frequencies and percentages. To ensure the reproducibility of the radiographic assessments, both intra-examiner and inter-examiner reliability were evaluated using a random subset of 50 sinuses. Agreement for categorical variables (e.g., SMT presence, PBL severity, and periapical lesions) was quantified using Cohen’s kappa statistics, with values above 0.80 indicating strong agreement.

### Multivariable modeling and internal validation

To maximize the utility of the large-scale dataset while accounting for the localized nature of odontogenic infections—where one sinus may exhibit membrane changes while the contralateral side remains healthy—data from both sinuses were included. Associations between SMT (> 2 mm) and odontogenic variables were initially evaluated using chi-square tests. To account for the clustering of bilateral sinuses within the same patient, multivariable logistic regression was performed using generalized estimating equations (GEE) with an exchangeable correlation structure. These analyses, along with the internal validation procedures, were conducted using Stata version 16.0 (StataCorp, College Station, TX, USA). The models were adjusted for age, sex, and smoking status, with PBL modeled as a categorical variable (coronal, middle, and apical thirds). Adjusted odds ratios (aORs) with 95% confidence intervals (CIs) were reported.

To assess the internal validity and stability of the multivariable GEE model, a bootstrapping procedure with 1,000 resamples was performed to estimate bias-corrected coefficients and CIs. Additionally, the goodness-of-fit of the GEE model was evaluated using the Quasi-likelihood under the Independence Model Criterion (QIC) and the Corrected QIC (QICC), which are appropriate for model selection in population-averaged models with clustered data.

### Development and performance of the COS

The COS was constructed by first scaling the β regression coefficients from the multivariable GEE model relative to the smallest coefficient (0.909). These raw weights were then iteratively optimized to reflect the biological gradient of periodontal destruction—where proximity to the sinus floor increases inflammatory risk—resulting in a 1-3-5 weighting for PBL and 1 point for binary endodontic factors. Score performance was evaluated based on both discrimination ability and calibration. Classification performance was assessed via receiver operating characteristic (ROC) curve analysis [18], with area under the curve (AUC) values and their 95% CIs calculated and compared. The ROC analysis and derived threshold describe classification performance within the same cross-sectional dataset and do not imply predictive capability or external validity. These ROC analyses were generated using R software (version 4.5.1) with the pROC package.

Model calibration was assessed through a calibration plot, calibration slope, and intercept to evaluate the agreement between estimated probabilities and observed SMT outcomes. A clinically relevant threshold for the COS was determined by maximizing the Youden index. All statistical tests were two-sided, and exact *p*-values were reported, with *p* < 0.05 considered statistically significant.

## Results

### Demographic and clinical characteristics

A total of 1,282 maxillary sinuses were initially screened, and after applying the exclusion criteria, 562 patients contributing 1,056 dentate maxillary sinuses were included in the final analytic sample. All included cases had complete clinical and radiographic data. As summarized in Table 1, the study population had a mean age of 53.5 ± 11.6 years (range, 21–86 years) with a slight female predominance (55.3%). A history of smoking was reported in 14.8% of the participants. At the sinus level, SMT (> 2 mm) was prevalent in 46.3% (489/1,056) of the sinuses. Among these, flat thickening was the most prevalent morphological pattern (74.0%), followed by polypoid (20.5%) and mucocele-like appearances (5.5%). Reliability analysis demonstrated strong consistency for all parameters, with intra-examiner and inter-examiner Cohen’s kappa coefficients ranging from 0.91 to 0.95 and 0.88–0.93, respectively, confirming the high reproducibility of the radiographic assessments.

**Table 1:** Demographic and radiographic characteristics of the study population and maxillary sinuses

**Variable**	
**Patient-level variables**	
Patients, n	562
Age, mean ± SD (range)	53.5 ± 11.6 (21–86)
Sex, n (%)	Female, 311 (55.3)
	Male, 251 (44.7)
Smoking status, n (%)	Yes, 83 (14.8)
	No, 479 (85.2)
**Sinus-level variables**	
Dentate maxillary sinuses, n	1056
SMT (> 2 mm), n (%)	489 (46.3)
No SMT, n (%)	567 (53.7)
**Morphology of membrane thickening**	
Flat, n (%)	362 (74.0)
Polypoid, n (%)	100 (20.5)
Mucocele-like, n (%)	27 (5.5)

Patient-level variables were summarized per individual (*n* = 562), whereas sinus-level variables and morphological patterns were summarized per maxillary sinus (*n* = 1056)

*SD* Standard deviation, *n* number, *SMT* Sinus membrane thickening

### Univariate associations with SMT

At the patient level (Table 2), male sex (*p* < 0.001) and smoking status (*p* = 0.019) were identified as significant contributors to SMT. At the sinus level (Table 3), univariate analyses demonstrated strong associations between SMT and all evaluated odontogenic variables. Sinuses adjacent to teeth with endodontic treatment history or periapical lesions exhibited a significantly higher prevalence of SMT compared to their respective counterparts (62.1% vs. 38.3% and 80.2% vs. 39.0%, respectively; *p* < 0.001). Furthermore, a clear dose-response relationship was observed between PBL severity and SMT prevalence, which increased progressively from 38.4% in the coronal-third group to 51.8% in the middle-third group, reaching 70.5% in the apical-third group (*p* < 0.001).

**Table 2:** Univariate associations between patient characteristics (*N* = 562) and SMT

Variables	SMT (*n* = 244)	Non-SMT (*n* = 318)	*p*-value
Age (years), mean ± SD	54.19 **±** 11.85	53.94 **±** 11.67	0.804
Sex, n (%)			
Female	106 (43.4)	205 (64.5)	< 0.001
Male	138 (56.6)	113 (35.5)	
Smoking status, n (%)			
Yes	49 (20.1)	34 (10.7)	0.019
No	195 (79.9)	284 (89.3)	

Patients were categorized into the SMT group if at least one maxillary sinus exhibited membrane thickening > 2 mm; otherwise, they were assigned to the Non-SMT group (both sinuses ≤ 2 mm). Statistical significance for continuous variables (age) was determined using the independent t-test, while categorical variables (sex and smoking status) were compared using the chi-square test. Significant at *p* < 0.05

*SD* Standard deviation, *n* number, *SMT* Sinus membrane thickening

**Table 3:** Univariate associations between odontogenic factors (*N* = 1,056) and SMT

Variables	SMT (*n* = 489)	Non-SMT (*n* = 567)	*p*-value
Endodontic treatment, *n* (%)			
Present	221 (45.2)	135 (23.8)	< 0.001
Absent	268 (54.8)	432 (76.2)	
Periapical lesion, n (%)			
Present	150 (30.7)	37 (6.5)	< 0.001
Absent	339 (69.3)	530 (93.5)	
Periodontal bone loss, n (%)			
Coronal third	226 (46.2)	363 (64.0)	
Middle third	184 (37.6)	171 (30.2)	< 0.001
Apical third	79 (16.2)	33 (5.8)	

Analyses for odontogenic factors were performed at the sinus level. Differences between groups were assessed using chi-square tests. Significant at *p* < 0.05

*n* number, *SMT* Sinus membrane thickening

### Multivariable analysis and model validation

**Fig. 3:**
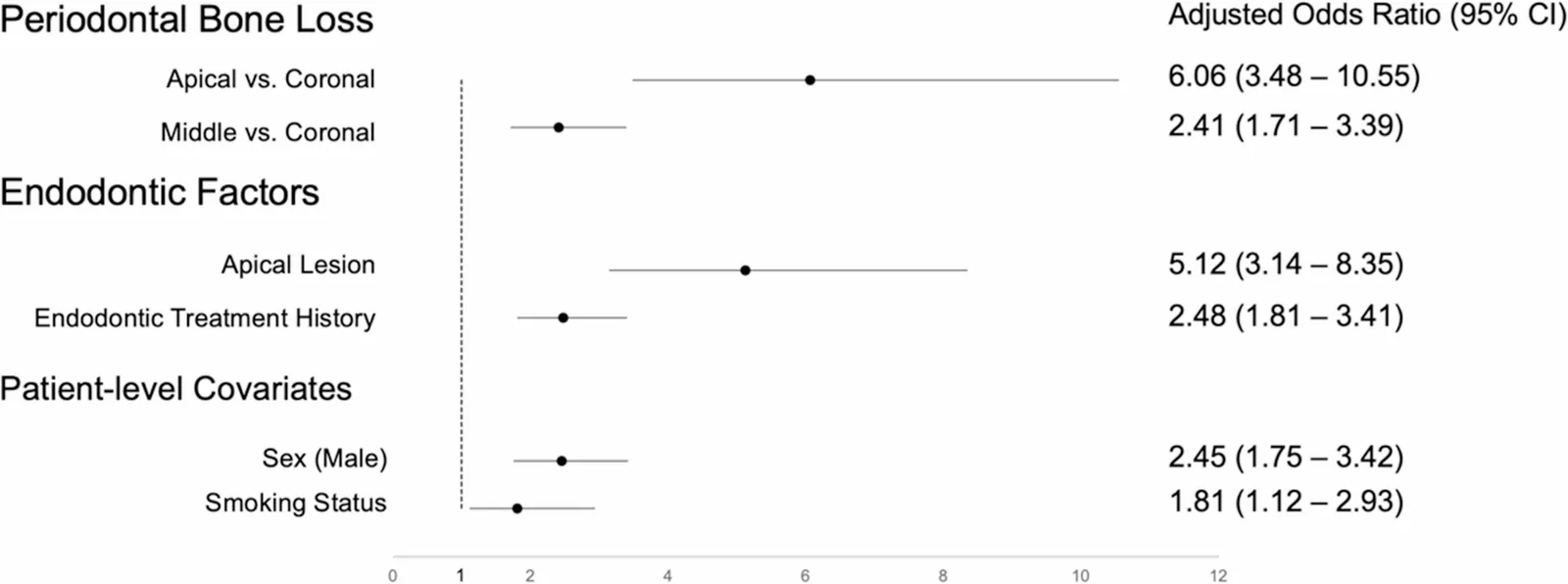
Forest plot of independent contributors associated with maxillary sinus membrane thickening. Adjusted odds ratios and 95% confidence intervals derived from the multivariable generalized estimating equation (GEE) model. The dashed vertical line represents the null effect (aOR = 1.0). All odontogenic and patient factors shown are mutually adjusted for age, sex, and smoking status

After adjusting for age, sex, and smoking status using GEE to account for patient-level clustering, all odontogenic factors remained independently associated with SMT (Table 4). Compared to the coronal-third reference, apical-third bone loss demonstrated a six-fold increase in the odds of SMT (aOR 6.06; 95% CI, 3.48–10.55, *p* < 0.001). The presence of a periapical lesion (aOR 5.12; 95% CI, 3.14–8.35, *p* < 0.001) and endodontic treatment history (aOR 2.48; 95% CI, 1.81–3.41, *p* < 0.001) were also potent independent factors (Fig. 3).

**Table 4:** Multivariable GEE logistic regression analysis of factors associated with SMT

Variable	Category	β	aOR	95% CI	*p*-value	Bootstrap		
						aOR	Normal-based 95% CI	*p*-value
Cluster	Left sinus	0.038	1.04	0.83–1.30	0.739	1.04	0.82–1.31	0.741
	Right sinus		1.00	(Reference)	–	1.00	(Reference)	–
Sex	Male	0.895	2.45	1.75–3.42	< 0.001	2.45	1.76–3.41	< 0.001
	Female		1.00	(Reference)	–	1.00	(Reference)	–
Smoking	Yes	0.594	1.81	1.12–2.93	0.015	1.81	1.03–3.18	0.039
	No		1.00	(Reference)	–	1.00	(Reference)	–
Endodontic treatment	Yes	0.909	2.48	1.81–3.41	< 0.001	2.48	1.78–3.44	< 0.001
	No		1.00	(Reference)	–	1.00	(Reference)	–
Periapical lesion	Yes	1.633	5.12	3.14–8.35	< 0.001	5.12	2.93–8.96	< 0.001
	No		1.00	(Reference)	–	1.00	(Reference)	–
Periodontal bone loss	Apical third	1.801	6.06	3.48–10.55	< 0.001	6.06	2.97–12.20	< 0.001
	Middle third	0.879	2.41	1.71–3.39	< 0.001	2.41	1.64–3.53	< 0.001
	Coronal third		1.00	(Reference)	–	1.00	(Reference)	–
Age (years)			0.99	0.98–1.00	0.149	0.99	0.98–1.00	0.129

Adjusted odds ratios were estimated using GEE to account for clustering of bilateral maxillary sinuses within the same patient. Models were mutually adjusted for all variables listed in the table, including age, sex, and smoking status. Model fit was assessed using the Quasi-likelihood under the Independence Model Criterion (QIC = 1257.022) and Corrected QIC (QICC = 1252.219). Internal validation was performed via bootstrapping with 1,000 resamples

*GEE* Generalized estimating equations, *SMT* Sinus membrane thickening, *β* Regression coefficient, *aOR* adjusted odds ratio, *CI* Confidence interval

The multivariable model demonstrated a stable fit, as indicated by a QIC of 1257.022 and a QICC of 1252.219. Internal validation via 1,000 bootstrap resamples confirmed the stability of these findings, with bias-corrected aORs remaining highly consistent with the original estimates (e.g., bootstrapped aOR 5.12 for periapical lesions and 6.06 for apical-third PBL), indicating that the model performance was not driven by outliers.

### Performance and classification ability of the COS

The COS was constructed by translating the β regression coefficients from the multivariable GEE model into an integer-based scoring system (Table 4). As detailed in the Methods, following an initial scaling based on the smallest β coefficient (0.909), the weights were calibrated to reflect the graded biological risk of various odontogenic conditions. During the development phase, various weighting schemes were iteratively evaluated to determine the optimal configuration. While a simple mathematical scaling of β coefficients suggested a 1-2-2 ratio, we found that expanding the PBL gradient to a 1-3-5 “spread” yielded superior classification performance (AUC 0.718) and a more favorable balance of sensitivity and specificity. Under this optimized framework, endodontic treatment history (β = 0.909) and periapical lesions (β = 1.633) were each assigned 1 point as binary indicators of inflammatory presence. PBL was weighted to reflect its non-linear biological association with SMT as bone loss encroaches upon the root apex: 1 point for coronal-third (β = reference), 3 points for middle-third (β = 0.879), and 5 points for apical-third involvement (β = 1.801). The resulting total score ranged from 1 to 7 points, with higher scores indicating a greater cumulative odontogenic inflammatory burden.

**Fig. 4:**
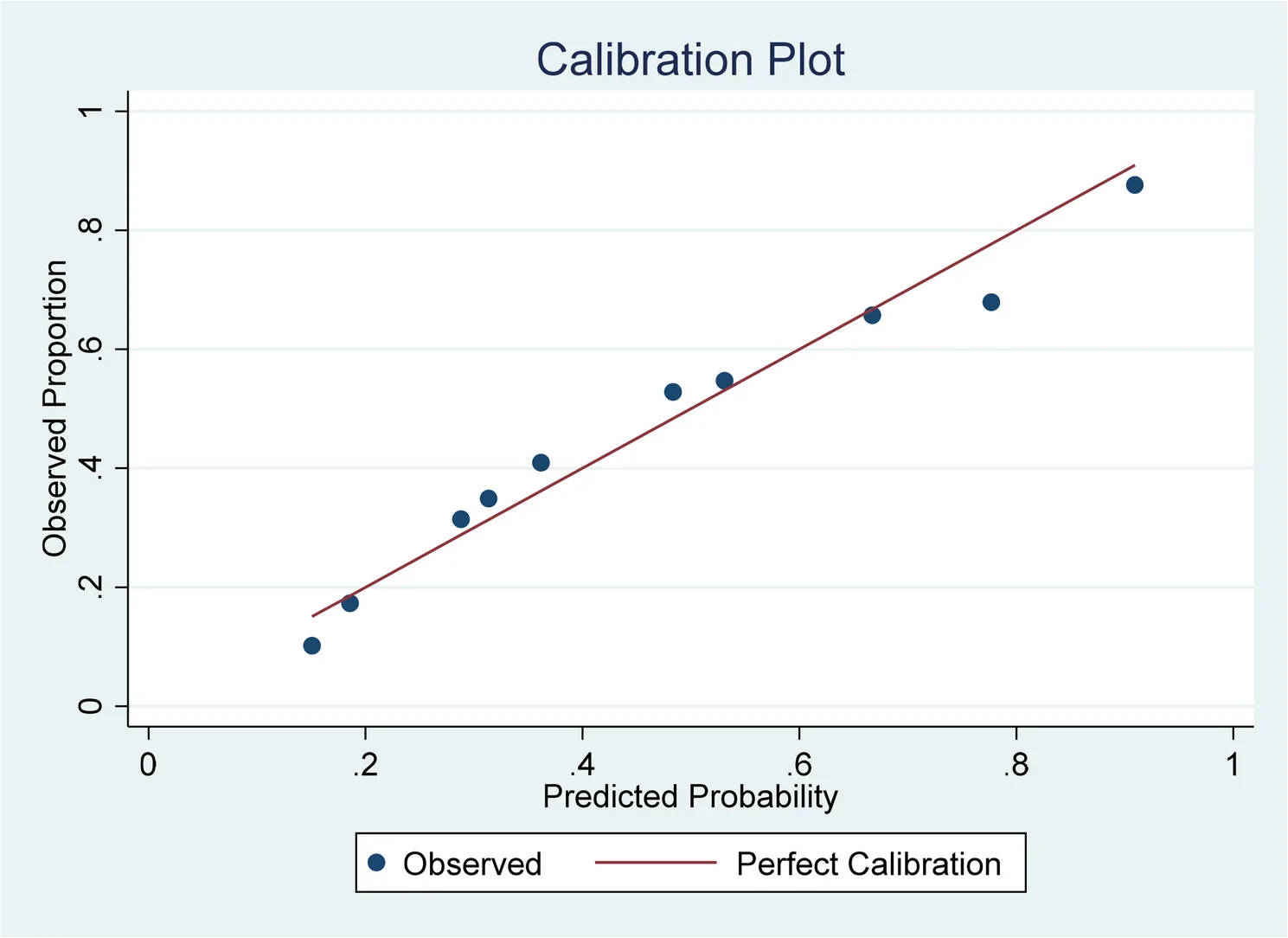
Calibration plot of the cumulative odontogenic score (COS). The plot illustrates the agreement between the estimated probability of sinus membrane thickening (SMT) and the observed proportions within the study sample. The x-axis represents the estimated probability calculated by the COS model, while the y-axis represents the actual observed frequency of SMT. The red solid line represents the ideal calibration (slope = 1, intercept = 0), where estimated and observed outcomes are identical. The proximity of the observed data points (blue dots) to the diagonal line indicates adequate model calibration within the study sample, supported by a calibration slope of 1.00 and a near-zero intercept (2.63 × 10^− 8^)

When modeled as a continuous variable, each one-point increase in the COS was associated with a 30.0% increase in the odds of SMT (OR 1.30; 95% CI, 1.22–1.38, *p* < 0.001). The COS demonstrated adequate calibration, with a calibration slope of 1.00 (95% CI: 0.86–1.14) and an intercept of 2.63 × 10^− 8^. The calibration plot (Fig. 4) showed a high level of agreement between the estimated probabilities and the observed SMT proportions.

**Fig. 5:**
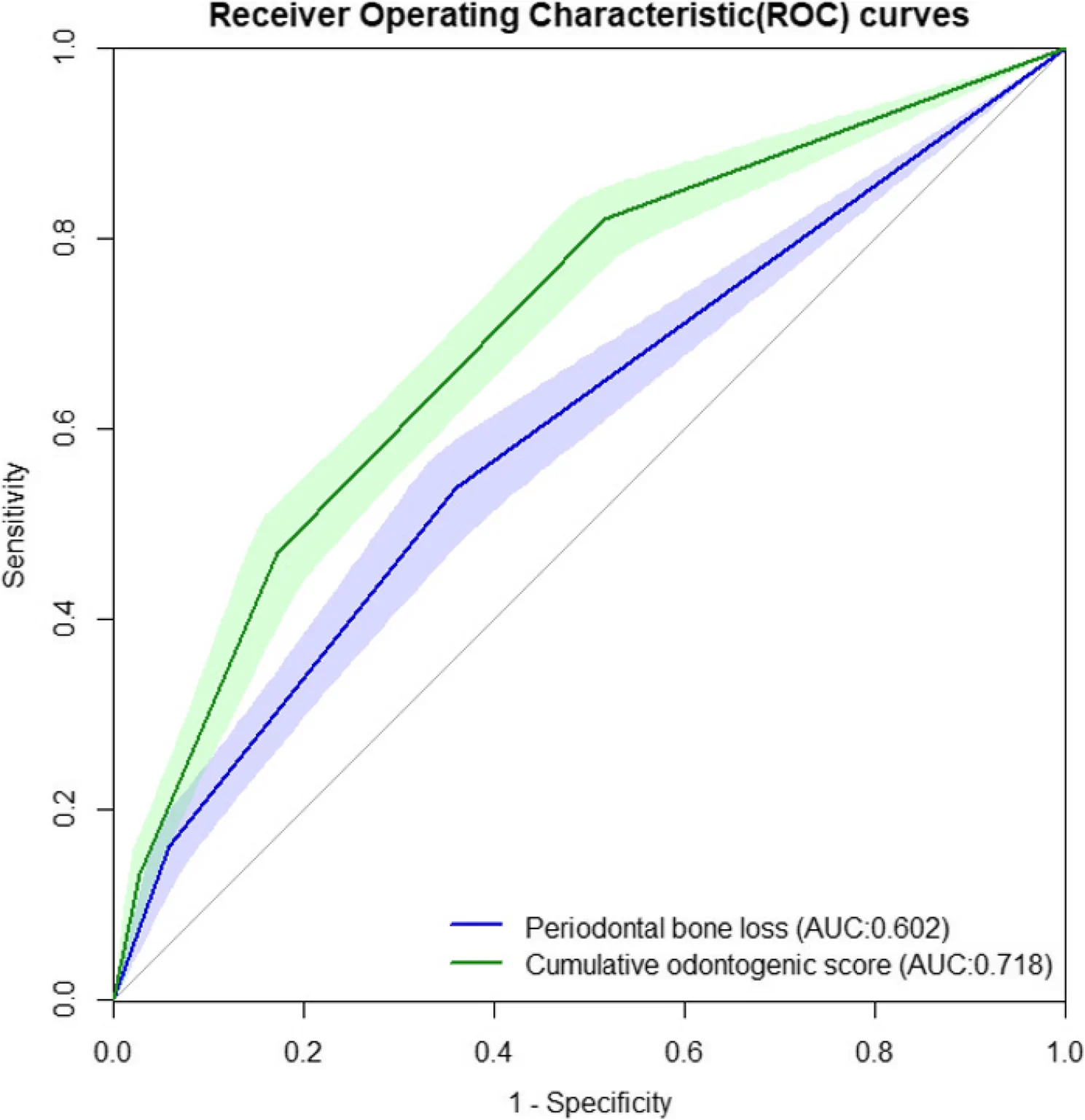
Classification performance of the cumulative odontogenic score (COS) within the dataset. Receiver operating characteristic (ROC) curves compare the classification performance of periodontal bone loss alone (blue line) and the cumulative odontogenic score (green line) for identifying sinus membrane thickening. The shaded areas represent the 95% confidence intervals (CIs) for each model, derived from bootstrapping procedures. The COS demonstrates significantly superior classification performance compared to the PBL-only model (area under the curve: 0.718 vs. 0.602; *p* < 0.001), indicating that integrating multiple odontogenic inflammatory sources enhances the identification of regional sinus membrane changes. The ROC analysis describes classification within the cross-sectional dataset and does not imply predictive capability

ROC curve analysis (Fig. 5) revealed that the COS (AUC 0.718; 95% CI, 0.688–0.749) provided significantly improved classification performance compared to PBL alone (AUC 0.602; 95% CI, 0.568–0.637; difference = 0.116, *p* < 0.001). The superior AUC of the composite COS compared to individual parameters was expected as it integrates the collective influence of multiple contributors within the same dataset. A COS threshold of > 3 points was identified as the clinically relevant cutoff for risk categorization. Using this threshold, the high-COS group exhibited nearly double the SMT prevalence of the low-COS group (59.9% vs. 30.5%; 340/568 vs. 149/488). This cutoff yielded a sensitivity of 69.5% (95% CI, 65.16–73.63) and a specificity of 59.8% (95% CI, 55.69–63.78), resulting in a positive likelihood ratio (LR+) of 1.73 and a negative likelihood ratio (LR-) of 0.51. Additionally, the positive predictive value (PPV) was 59.9% (95% CI, 55.74–63.87) and the negative predictive value (NPV) was 69.5% (95% CI, 65.12–73.59), with an overall classification accuracy of 64.3%.

## Discussion

The present study characterizes the cumulative odontogenic influence on the maxillary sinus membrane, transitioning from a localized dental perspective to a regional inflammatory framework. Our findings reveal an SMT (> 2 mm) prevalence of 46.3%, consistent with prior CBCT-based observations [4, 6, 9, 17, 19]. Given that preexisting SMT is linked to impaired sinus ventilation [10], higher perforation risks during sinus floor elevation [20, 21], and altered graft healing [22], identifying the odontogenic drivers of these sinus membrane changes is paramount for mitigating surgical risks in the posterior maxilla.

Consistent with the “perio-antral” link, multivariable analysis identified PBL severity, periapical lesions, and endodontic treatment history as independent contributors to SMT. The potent association with periapical pathology (aOR 5.12) underscores the sinus membrane’s role as a biological sensor for periapical inflammation [7, 10], likely due to the diffusion of mediators through vascular channels or trabecular porosities [6, 23]. Furthermore, the graded relationship observed between PBL severity and SMT prevalence confirms that chronic periodontal inflammation exerts a persistent, additive burden on the maxillary sinus membrane [8, 9, 24, 25].

A primary contribution of this study is the introduction of the COS, which integrates these disparate dental pathologies into a unified, integer-based framework. While the improved classification performance of the composite COS (AUC 0.718) over individual parameters like PBL (AUC 0.602) is mathematically anticipated, these results emphasize that relying solely on single factors may provide an incomplete assessment of the regional dental-sinus relationship. The additive nature of the COS reflects the cumulative inflammatory load associated with concurrent infections, thereby serving as an interdisciplinary bridge to ensure that dental inflammatory reservoirs are addressed prior to elective sinus interventions. Furthermore, assigning a baseline score of 1 for all dentate sinuses acknowledges that the presence of dentition itself represents a distinct physiological context compared to the edentulous state [7, 8], thereby providing a refined baseline for evaluating cumulative odontogenic effects.

The proposed categorization threshold (COS > 3) effectively identifies a high-COS category where the prevalence of SMT is nearly doubled (59.9% vs. 30.5%). This threshold can be reached either through a combination of moderate factors—such as middle-third PBL (3 points) alongside a periapical lesion (1 point)—or by a single severe condition, such as apical-third PBL (5 points). While the COS was derived from 3D measurements, its preliminary clinical application to 2D radiographs is proposed as a conceptual risk categorization strategy. We explicitly acknowledge that applying this framework to 2D imaging—which involves inherent superimposition—remains a theoretical recommendation that has not been formally validated.

**Fig. 6:**
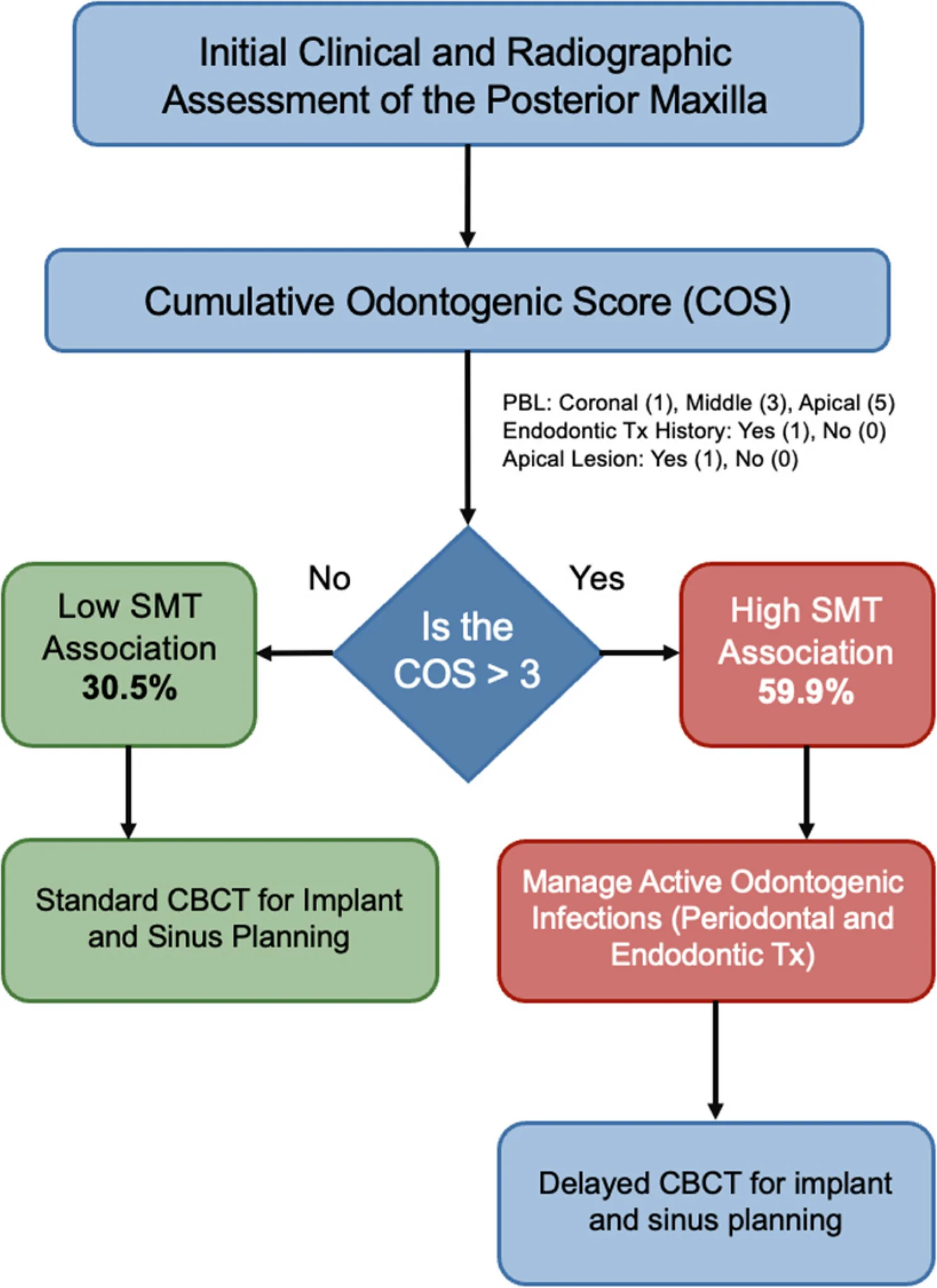
Proposed conceptual framework for interdisciplinary preoperative evaluation. This flowchart provides a conceptual illustration of the interdisciplinary management of patients in the posterior maxilla based on the cumulative odontogenic score (COS). A threshold of COS > 3 identifies a high-COS category, potentially guiding the evaluation of active inflammatory reservoirs prior to definitive surgical intervention. By addressing odontogenic infections prior to definitive imaging, this proposed approach adheres to the ALARA principle by minimizing the need for repetitive CBCT scans after membrane resolution. This framework serves as a hypothesis-generating model for future clinical validation. CBCT: cone-beam computed tomography; COS: cumulative odontogenic score; PBL: periodontal bone loss; SMT: sinus membrane thickening; Tx: treatment; ALARA: As Low As Reasonably Achievable

Despite these 2D limitations, identifying high-COS cases during initial evaluation provides a critical opportunity for early intervention. Based on this framework, we propose a conceptual “resolution-first” pathway (Fig. 6) to guide interdisciplinary clinical management. By addressing active odontogenic infections before finalizing the CBCT-based treatment plan, clinicians might establish a more stable sinus environment, potentially optimizing surgical conditions and adhering to the ALARA (As Low As Reasonably Achievable) principle [26] by minimizing the need for repetitive imaging after infection resolution. Furthermore, this approach functions as a diagnostic filter: SMT resolution following dental intervention would support an odontogenic etiology, whereas persistent thickening raises suspicion of non-odontogenic origins, necessitating referral to an otolaryngologist for further evaluation. This proposed workflow serves as a hypothesis-generating model for future prospective validation.

Several limitations warrant consideration. First, the retrospective cross-sectional design precludes establishing temporal or causal relationships. It is important to emphasize that the ROC analysis and derived threshold describe classification performance within the same cross-sectional dataset and do not imply predictive capability or external validity. Consequently, the COS should be interpreted as a classification framework for concurrent associations rather than a definitive diagnostic or predictive engine. Second, while the COS underwent systematic internal validation via bootstrapping (1,000 resamples) and demonstrated favorable calibration (slope = 1.00), it was developed within a single-center Taiwanese population consisting of implant-seeking patients with preserved dentition. Future multicenter studies are essential to confirm its external generalizability. Third, radiographic SMT (> 2 mm) lacks clinical specificity, as it may represent incidental, physiological, or allergic responses rather than active odontogenic pathology. Although we excluded overt sinonasal diseases, subclinical non-odontogenic confounders [5, 27] cannot be entirely ruled out. The moderate classification performance of the COS is therefore expected, as it is intended to complement, not replace, comprehensive clinical judgment. Finally, while our analysis identified male sex and smoking as significant contributors to SMT, we did not account for granular data such as smoking intensity (pack-years) or duration of cessation. These systemic factors may introduce residual confounding, and future studies focusing on their dose-response relationships with sinus health are warranted to refine the COS.

## Conclusions

Multiple odontogenic conditions—including PBL, endodontic treatment history, and periapical lesions—demonstrate a cumulative association with SMT. Integrating these variables into a COS provides a more structured characterization of the dental-sinus association than traditional individual assessments. A COS > 3 serves as an initial clinical threshold for preoperative risk categorization. This internally validated framework facilitates more standardized interdisciplinary evaluation and supports preliminary risk characterization in the posterior maxilla, although it remains a cross-sectional classification tool pending further external validation.

## Data Availability

The datasets analyzed during the current study are available from the corresponding author on reasonable request.

## Abbreviations

ALARA: As Low As Reasonably Achievable
aOR: Adjusted Odds Ratio
AUC: Area Under the Curve
CBCT: Cone-beam Computed Tomography
CI: Confidence Interval
COS: Cumulative Odontogenic Score
GEE: Generalized Estimating Equations
LR+: Positive likelihood ratio
LR-: Negative likelihood ratio
NPV: Negative Predictive Value
PBL: Periodontal Bone Loss
PPV: Positive Predictive Value
QIC: Quasi-likelihood under the Independence Model Criterion
QICC: Corrected Quasi-likelihood under the Independence Model Criterion
ROC: Receiver Operating Characteristic
SMT: Sinus Membrane Thickening

## References

[1] Papapanou PN, et al. Periodontitis: Consensus report of workgroup 2 of the 2017 World Workshop on the Classification of Periodontal and Peri-Implant Diseases and Conditions. J Periodontol. 2018;89(Suppl 1):S173–82. 29926951 10.1002/JPER.17-0721

[2] Tonetti MS, Greenwell H, Kornman KS. Staging and grading of periodontitis: Framework and proposal of a new classification and case definition. J Periodontol. 2018;89(Suppl 1):S159–72. 29926952 10.1002/JPER.18-0006

[3] Kornman KS, Papapanou PN. Clinical application of the new classification of periodontal diseases: Ground rules, clarifications and gray zones. J Periodontol. 2020;91(3):352–60. 31891194 10.1002/JPER.19-0557

[4] Block MS, Dastoury K. Prevalence of sinus membrane thickening and association with unhealthy teeth: a retrospective review of 831 consecutive patients with 1,662 cone-beam scans. J Oral Maxillofac Surg. 2014;72(12):2454–60. 25236817 10.1016/j.joms.2014.06.442

[5] Vallo J, et al. Prevalence of mucosal abnormalities of the maxillary sinus and their relationship to dental disease in panoramic radiography: results from the Health 2000 Health Examination Survey. Oral Surg Oral Med Oral Pathol Oral Radiol Endod. 2010;109(3):e80–7. 20219592 10.1016/j.tripleo.2009.10.031

[6] Janner SF, et al. Characteristics and dimensions of the Schneiderian membrane: a radiographic analysis using cone beam computed tomography in patients referred for dental implant surgery in the posterior maxilla. Clin Oral Implants Res. 2011;22(12):1446–53. 21426404 10.1111/j.1600-0501.2010.02140.x

[7] Lu Y, et al. Associations between maxillary sinus mucosal thickening and apical periodontitis using cone-beam computed tomography scanning: a retrospective study. J Endod. 2012;38(8):1069–74. 22794207 10.1016/j.joen.2012.04.027

[8] Phothikhun S, et al. Cone-beam computed tomographic evidence of the association between periodontal bone loss and mucosal thickening of the maxillary sinus. J Periodontol. 2012;83(5):557–64. 21910593 10.1902/jop.2011.110376

[9] Ren S, et al. Significance of maxillary sinus mucosal thickening in patients with periodontal disease. Int Dent J. 2015;65(6):303–10. 26453062 10.1111/idj.12186 PMC9376544

[10] Shanbhag S, et al. Cone-beam computed tomographic analysis of sinus membrane thickness, ostium patency, and residual ridge heights in the posterior maxilla: implications for sinus floor elevation. Clin Oral Implants Res. 2014;25(6):755–60. 23560797 10.1111/clr.12168

[11] Herrera D, et al. Acute periodontal lesions (periodontal abscesses and necrotizing periodontal diseases) and endo-periodontal lesions. J Periodontol. 2018;89(Suppl 1):S85–102. 29926942 10.1002/JPER.16-0642

[12] Rotstein I. J.H. <>Simon 2004 Diagnosis, prognosis and decision-making in the treatment of combined periodontal-endodontic lesions. Periodontol 2000 34 p165–203. 10.1046/j.0906-6713.2003.003431.x 14717862

[13] Siqueira JF Jr., Rocas IN. Clinical implications and microbiology of bacterial persistence after treatment procedures. J Endod. 2008;34(11):1291–e13013. 18928835 10.1016/j.joen.2008.07.028

[14] Janner SFM, et al. Sinus floor elevation or referral for further diagnosis and therapy: A comparison of maxillary sinus assessment by ENT specialists and dentists using cone beam computed tomography. Clin Oral Implants Res. 2020;31(5):463–75. 31991010 10.1111/clr.13582

[15] von Elm E, et al. The Strengthening the Reporting of Observational Studies in Epidemiology (STROBE) statement: guidelines for reporting observational studies. Lancet. 2007;370(9596):1453–7. 18064739 10.1016/S0140-6736(07)61602-X

[16] Chen HH, et al. Anatomical characteristics of maxillary sinus antroliths and their influence on sinus membrane thickness: a retrospective cone beam computed tomography analysis. Int J Oral Maxillofac Surg. 2021;50(8):1107–12. 33431227 10.1016/j.ijom.2020.12.010

[17] Schneider AC, et al. Characteristics and dimensions of the sinus membrane in patients referred for single-implant treatment in the posterior maxilla: a cone beam computed tomographic analysis. Int J Oral Maxillofac Implants. 2013;28(2):587–96. 23527364 10.11607/jomi.3007

[18] Hanley JA, McNeil BJ. A method of comparing the areas under receiver operating characteristic curves derived from the same cases. Radiology. 1983;148(3):839–43. 6878708 10.1148/radiology.148.3.6878708

[19] Drumond JP, et al. Evaluation of the Prevalence of Maxillary Sinuses Abnormalities through Spiral Computed Tomography (CT). Int Arch Otorhinolaryngol. 2017;21(2):126–33. 28382118 10.1055/s-0036-1593834 PMC5375949

[20] Lin YH, et al. The influence of sinus membrane thickness upon membrane perforation during lateral window sinus augmentation. Clin Oral Implants Res. 2016;27(5):612–7. 26076580 10.1111/clr.12646

[21] Wen SC, et al. The influence of sinus membrane thickness upon membrane perforation during transcrestal sinus lift procedure. Clin Oral Implants Res. 2015;26(10):1158–64. 24891094 10.1111/clr.12429

[22] Chen HH, et al. Impact of Membrane Thickening and Perforation on Graft Remodeling After Osteotome Sinus Elevation: A Retrospective Study. Clin Implant Dent Relat Res. 2025;27(6):e70103. 41292419 10.1111/cid.70103

[23] Bornstein MM, et al. Characteristics and dimensions of the Schneiderian membrane and apical bone in maxillary molars referred for apical surgery: a comparative radiographic analysis using limited cone beam computed tomography. J Endod. 2012;38(1):51–7. 22152620 10.1016/j.joen.2011.09.023

[24] Eggmann F, et al. Do periapical and periodontal pathologies affect Schneiderian membrane appearance? Systematic review of studies using cone-beam computed tomography. Clin Oral Investig. 2017;21(5):1611–30. 27585589 10.1007/s00784-016-1944-7

[25] Sheikhi M, Pozve NJ, Khorrami L. Using cone beam computed tomography to detect the relationship between the periodontal bone loss and mucosal thickening of the maxillary sinus. Dent Res J (Isfahan). 2014;11(4):495–501. 25225564 PMC4163829

[26] The 2007 Recommendations of the International Commission on Radiological Protection. ICRP publication 103. Ann ICRP, 2007. 37(2–4): pp. 1–332. 10.1016/j.icrp.2007.10.003 18082557

[27] Ritter L, et al. Prevalence of pathologic findings in the maxillary sinus in cone-beam computerized tomography. Oral Surg Oral Med Oral Pathol Oral Radiol Endod. 2011;111(5):634–40. 21444226 10.1016/j.tripleo.2010.12.007

